# Lower Dietary Intake of Plant Protein Is Associated with Genetic Risk of Diabetes-Related Traits in Urban Asian Indian Adults

**DOI:** 10.3390/nu13093064

**Published:** 2021-08-31

**Authors:** Sooad Alsulami, Dhanasekaran Bodhini, Vasudevan Sudha, Coimbatore Subramanian Shanthi Rani, Rajendra Pradeepa, Ranjit Mohan Anjana, Venkatesan Radha, Julie A. Lovegrove, Rajagopal Gayathri, Viswanathan Mohan, Karani Santhanakrishnan Vimaleswaran

**Affiliations:** 1 Hugh Sinclair Unit of Human Nutrition, Department of Food and Nutritional Sciences, University of Reading, Reading RG6 6DZ, UK; s.alsulami@student.reading.ac.uk (S.A.); j.a.lovegrove@reading.ac.uk (J.A.L.); 2 Department of Clinical Nutrition, Faculty of Applied Medical Sciences, King Abdulaziz University, Jeddah 21589, Saudi Arabia; 3 Department of Molecular Genetics, Madras Diabetes Research Foundation, Chennai 603103, India; bodhinid@gmail.com (D.B.); radharv@yahoo.co.in (V.R.); 4 Department of Foods, Nutrition and Dietetics Research, Madras Diabetes Research Foundation, Chennai 600086, India; s2r_7@mdrf.in (V.S.); gayathri@mdrf.in (R.G.); 5 Department of Clinical Epidemiology, Madras Diabetes Research Foundation, Chennai 600086, India; kshan_rany@yahoo.com; 6 Department of Diabetology, Madras Diabetes Research Foundation & Dr. Mohan’s Diabetes Specialities Centre, WHO Collaborating Centre for Non-Communicable Diseases Prevention and Control, ICMR Centre for Advanced Research on Diabetes, Gopalapuram, Chennai 600086, India; guhapradeepa@gmail.com (R.P.); dranjana@drmohans.com (R.M.A.); drmohans@diabetes.ind.in (V.M.); 7 The Institute for Food, Nutrition, and Health (IFNH), University of Reading, Reading RG6 6AP, UK

**Keywords:** genetic risk score, metabolic traits, urban Asian Indian, dietary protein intake, gene–diet interaction, T2D

## Abstract

The increasing prevalence of type 2 diabetes among South Asians is caused by a complex interplay between environmental and genetic factors. We aimed to examine the impact of dietary and genetic factors on metabolic traits in 1062 Asian Indians. Dietary assessment was performed using a validated semi-quantitative food frequency questionnaire. Seven single nucleotide polymorphisms (SNPs) from the Transcription factor 7-like 2 and fat mass and obesity-associated genes were used to construct two metabolic genetic risk scores (GRS): 7-SNP and 3-SNP GRSs. Both 7-SNP GRS and 3-SNP GRS were associated with a higher risk of T2D (*p* = 0.0000134 and 0.008, respectively). The 3-SNP GRS was associated with higher waist circumference (*p* = 0.010), fasting plasma glucose (FPG) (*p* = 0.002) and glycated haemoglobin (HbA1c) (*p* = 0.000066). There were significant interactions between 3-SNP GRS and protein intake (% of total energy intake) on FPG (P_interaction_ = 0.011) and HbA1c (P_interaction_ = 0.007), where among individuals with lower plant protein intake (<39 g/day) and those with >1 risk allele had higher FPG (*p* = 0.001) and HbA1c (*p* = 0.00006) than individuals with ≤1 risk allele. Our findings suggest that lower plant protein intake may be a contributor to the increased ethnic susceptibility to diabetes described in Asian Indians. Randomised clinical trials with increased plant protein in the diets of this population are needed to see whether the reduction of diabetes risk occurs in individuals with prediabetes.

## 1. Introduction

South Asian populations have a 50% higher risk of type 2 diabetes (T2D) than other populations [1,2] and this has significant implications, as patients with T2D have a 2–4 times increased risk of cardiovascular diseases [1]. The Asian Indian population have a unique phenotype characterised by abdominal and truncal adiposity, as indicated by larger waist to hip ratios and waist circumference (WC), higher concentrations of plasma insulin, greater insulin resistance, impaired function of pancreatic β-cell and a genetic susceptibility to diabetes, which ultimately leads to significantly increased diabetes risk [3,4,5]. The burden of T2D is increasing globally, with India being a major contributor to the worldwide burden [6]. The number of diabetic individuals in India rose from 26.0 million in 1990 to 65.0 million in 2016 [7].

The increasing prevalence of T2D among Asian Indians is caused by a complex interplay between environmental and genetic factors, including urbanisation, which plays a large role [8,9,10]. Urbanisation in India is associated with increased consumption of processed foods and dietary fats, decreased level of physical activity and increased mental stress, amplifying the effects of abdominal obesity and insulin resistance [4,5,11]. Furthermore, the urban areas in India reported higher intake of protein from pulses and animal sources (including meat, fish, eggs and milk) than rural areas [12]. Several large longitudinal studies showed that the intake of animal protein was significantly associated with the risk of T2D [13,14,15,16,17]. In the context of rapid urbanisation and nutrition transition, interactions between Westernised diet, lifestyle and genetic factors have further escalated T2D prevalence in Asia [18,19]. In South Asians, several single nucleotide polymorphisms (SNPs) have been associated with adiposity [20,21,22,23], insulin resistance [24], pancreatic β-cell function [20,25,26] and T2D [20,22,23,26,27]. The fat mass and obesity-associated *(FTO)* gene has been recognised as one of the strongest obesity-related genes. The *FTO* SNPs, rs1588413, rs9939609 and rs8050136, have been shown to increase obesity risk by 1.27, 1.15 and 2.06 times among Indians, respectively [22,28]. Studies have reported strong associations of the Transcription factor 7-like 2 *(TCF7L2)* SNPs, rs7903146 and rs12255372, with T2D risk in Asian Indians [29,30,31]. To date, evidence has identified 243 genetic loci to be associated with T2D risk in South Asians, East Asians, Europeans, African Americans and Hispanics [32,33,34,35]. Single genetic variants have only a small to moderate effect on disease risk, thus combining effects of several SNPs into a genetic risk score (GRS) is required for better detection of individuals with high risk of diabetes [36].

Genome-wide association studies (GWAS) have discovered a large number of genetic variants associated with metabolic diseases and related traits; however, these SNPs describe only a small proportion of estimated heritability. Risk prediction of metabolic diseases is complicated by interactions between dietary and genetic factors, which may partly explain the missing heritability of diseases [37]. Investigating gene–diet interaction is important in understanding pathophysiology of metabolic diseases, which can lead to the development of ‘personalised’ nutrition focusing on tailoring dietary interventions according to individual genotypic makeup to prevent and treat metabolic diseases [38,39]. The effect of genetic factors on metabolic traits have been shown to be modified by dietary intake in several populations [40,41,42,43,44]. However, studies investigating GRS–diet interaction in the Indian population are still sparse. To help fill this gap in knowledge, we assessed the combined effect of seven genetic variants, as a GRS, on T2D and metabolic traits, and the extent to which dietary intake can influence these genetic associations among 1062 urban Asian Indians.

## 2. Methods

### 2.1. Study Participants

The present study included individuals from the urban area of the Chennai Urban Rural Epidemiology Study (CURES), which is a cross-sectional epidemiological study performed on a representative sample of Chennai city (formerly Madras) in southern India. The design and procedures of the CURES have been explained in detail previously [45]. In phase 1, a total of 26,001 adult subjects, of which 1529 were ‘self-reported’ or ‘known diabetic’ individuals, were recruited using a method of systematic random sampling. In phase 2, diabetic individuals were invited to the study centre for further investigation, of whom 1382 responded. In phase 3, every 10th individual of the total sample (*n* = 26,001 subjects), excluding individuals with self-reported diabetes, were screened using an oral glucose tolerance test (OGTT). Individuals with fasting plasma glucose (FPG) < 5.6 mmol/L (100 mg/dL) and 2 h plasma glucose value of 7.8 mmol/L (140 mg/dL) were defined as having normal glucose tolerance (NGT) [46]. Those who had 2 h plasma glucose value of 11.1 mmol/L (200 mg/dL) were categorised as ‘newly detected diabetic subjects’ (*n* = 222) (Figure S1). The total sample of present study is 1062 individuals; the NGT individuals were chosen from Phase 3 (*n* = 496) and T2D individuals were chosen from Phase 2 and Phase 3 of the CURES (*n* = 566). The study was approved by the Madras Diabetes Research Foundation Institutional Ethics Committee and written informed consent was obtained from all study participants.

### 2.2. Anthropometric and Biochemical Measurements

Anthropometric variables including WC, weight and height were measured using standardised methods. The body mass index (BMI) was calculated with the formula of weight (in kilograms) divided by the square of height (in metres), with obesity being defined as BMI ≥ 25 according to World Health Organisation Asia Pacific Guidelines for Asians [47].

Biochemical tests were carried out using a Hitachi-912 Auto Analyzer (Hitachi, Mannheim, Germany), with kits provided by Roche Diagnostics (Mannheim). Glycated haemoglobin (HbA1c) was measured using high-performance liquid chromatography on a Variant machine (Bio-Rad, Hercules, CA, USA). FPG and serum insulin were measured using glucose oxidase-peroxidase and an enzyme-linked immunosorbent assay (Dako, Glostrup, Denmark), respectively.

### 2.3. Dietary Assessments

Participants’ habitual food intake over the previous year was measured using a validated semi-quantitative food frequency questionnaire (FFQ) administered by an interviewer [48]. The FFQ consists of 222 food items and individuals were asked to estimate the usual portion size and frequency (number of times per day, week, month or year/never) of food items listed in the FFQ. Participants were shown common household measures and photographic atlas of different sizes of fruits to help them in estimating portion sizes. The EpiNu^®^ software was used to analyse the recorded data and estimate the intake of energy and macronutrients. The reported intake of various food groups was also estimated. The EpiNu software also provided the source of protein from various food groups. Animal protein intake was summed up using protein intake (g/day from FFQ) from animal food groups such as meat, poultry, fish, egg and dairy products. Similarly, plant protein intake was estimated from food groups such as cereals, millets, pulses and legumes, tubers, nuts, oilseeds, vegetables and fruits. In addition, dairy protein was estimated separately using dairy products such as milk products and fermented and unfermented milk.

### 2.4. SNP Selection and GRS Construction

A total of 7 metabolic disease-associated SNPs which have been extensively studied in various populations, including Asian Indians, were selected for the study [20,21,22,23,24,25,26,27,29]. The selected SNPs included *TCF7L2* SNPs, rs12255372 and rs7903146, and *FTO* SNPs, rs8050136, rs918031, rs1588413, rs7193144 and rs1076023. Details regarding these SNPs are summarised in Table S1. Each SNP was coded with the expected number of metabolic diseases-associated risk alleles. Consistent with previous studies [41,49,50], we used an unweighted method to construct the GRSs by summing the number of risk alleles of each SNP for each participant. The seven SNPs were used to generate a 7-SNP GRS that ranges from 1 to 11 risk alleles. The GRS was divided into 2 categories according to the median number of risk alleles: “GRS < 6 risk alleles” and “GRS ≥ 6 risk alleles”, indicating individuals with lower and higher risk alleles of the SNPs, respectively. In addition, we constructed a GRS of 3 SNPs (*FTO*SNP rs8050136 and *TCF7L2*SNPs rs12255372 and rs7903146) that have shown consistent associations with metabolic disease-related outcomes across various ethnicities, including Asians [51,52,53,54]. The 3-SNP GRS ranges from 0 to 6 risk alleles and was divided into 2 categories according to the median number of risk alleles: “GRS ≤ 1 risk allele” group and “GRS > 1 risk allele” group, indicating individuals with lower and higher risk alleles of the SNPs, respectively.

### 2.5. Genotyping

The genotyping methodologies have been previously published [22,30]. The phenol-chloroform technique was used to extract DNA from whole blood. Genotyping was performed using restriction fragment length polymorphism and confirmed by direct sequencing in which duplicate samples (*n* = 200; 20%) were genotyped with 100% concordance, suggesting high genotyping accuracy.

### 2.6. Statistical Analysis

Descriptive statistics of continuous variables are provided as means with standard deviations (SDs) and compared between T2D and controls using an independent sample *t*-test. Normality tests were performed and variables with no-normal distribution were log transformed. For each individual SNP, genotype counts were assessed for Hardy–Weinberg equilibrium (HWE) using a goodness-of-fit chi-square test. As shown in Table S1, all SNPs were in HWE (*p* > 0.092, for all comparisons). General linear models were utilised to analyse the main associations of the GRS with metabolic traits. Interactions of the GRS with dietary intake were investigated by including the interaction term (GRS*dietary intake) in the models. Furthermore, significant interactions with protein intake were analysed in more depth according to dietary sources of protein (animal and plant protein), where individuals were classified into two groups according to the sample median intake of plant (39 g/day) and animal protein (19 g/day): below and above median groups. Individuals who consumed below the median were categorised as those who had lower intakes of plant and animal protein, respectively, whereas individuals who consumed above the median were categorised as those who had higher intakes of plant and animal protein, respectively. Dietary intakes as percentage of total energy intake (TEI) included intake of protein, carbohydrate and fat. Models were adjusted for sex, age, T2D, anti-diabetic medication and BMI (when BMI is not an outcome). Furthermore, as part of the sensitivity analysis, we further adjusted for duration of diabetes, dairy protein intake, physical activity level, smoking, alcohol consumption and fibre intake. Statistical analyses were carried out using Statistical Package for the Social Sciences (SPSS) software (version 24; SPSS Inc., Chicago, IL, USA), with a significance level of 0.05.

## 3. Results

### 3.1. Characteristics of Study Participants

As shown in Table 1, individuals with T2D were significantly older and had higher BMI, WC, HbA1c, FPG and insulin, compared to individuals with NGT (*p* < 0.05 for all). Moreover, diabetic individuals had significantly higher intakes of total protein and carbohydrate than individuals with NGT (*p* < 0.05 for all).

### 3.2. Association between Metabolic GRS and Metabolic Traits

After adjusting for the potential confounders there were no significant associations between the 7-SNP GRS and metabolic traits (Table 2).

In the 3-SNP GRS analysis, significant associations were found with WC (*p* = 0.010), FPG (*p* = 0.002) and HbA1c (*p* = 0.000066), where individuals with >1 risk allele had higher WC, FPG and HbA1c compared to individuals with ≤1 risk allele (Table 2). Both 7-SNP GRS and 3-SNP GRS were associated with a higher risk of T2D (*p* = 0.0000134 and 0.008, respectively) (Table 3).

### 3.3. Interaction of 7-SNP and 3-SNP GRSs with Dietary Factors on Metabolic Traits

**Figure 1:**
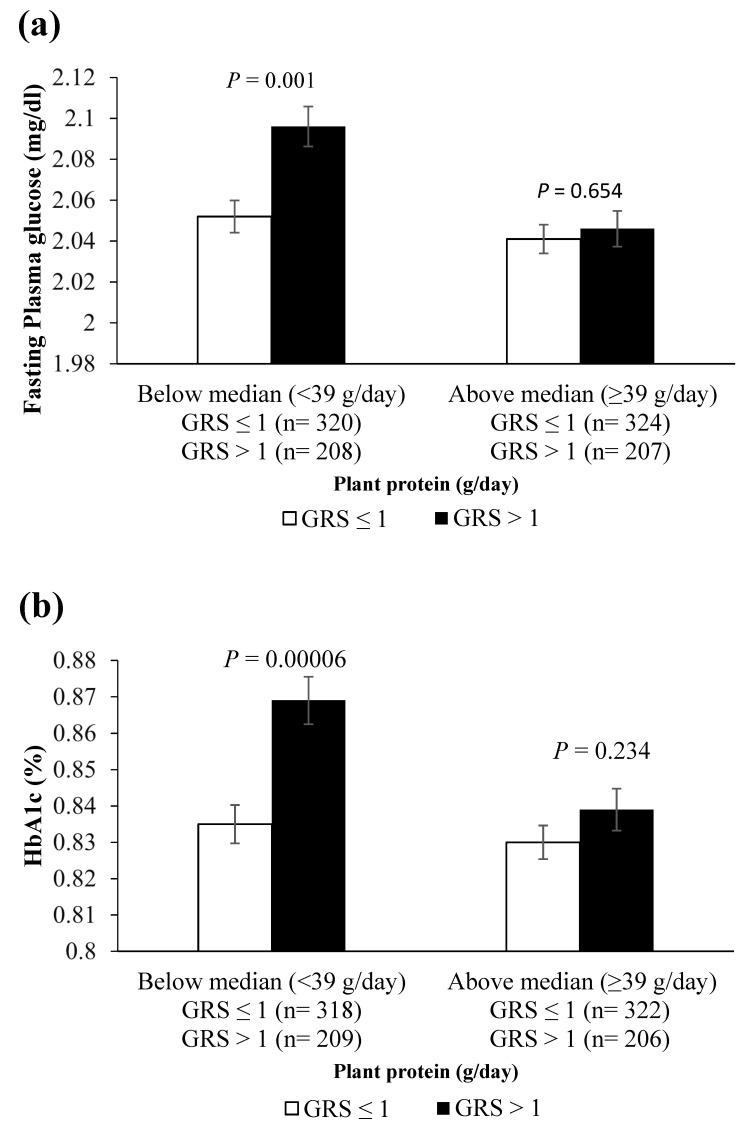
Interaction between 3-SNP GRS and plant protein intake on fasting plasma glucose and glycated haemoglobin. White bars refer to individuals with GRS ≤ 1 risk allele; the black bars refer to individuals with GRS > 1 risk allele. (**a**) Individuals with >1 risk allele had a significantly higher FPG compared to those with ≤1 risk allele, among those with lower intake of plant protein (<39 g/day) (*p* = 0.001). (**b**) Individuals with >1 risk allele had a significantly higher HbA1c compared to those with ≤1 risk allele, among those with lower intake of plant protein (<39 g/day) (*p* = 0.00006). *p* values were adjusted for age, sex, T2D, BMI, anti-diabetic medication, total fat intake (%) and TEI. Abbreviations: GRS, genetic risk score; FPG, fasting plasma glucose; HbA1c, glycated haemoglobin; TEI, total energy intake.

As shown in Table 4, there were significant interactions between the 3-SNP GRS and total protein intake (% of TEI) on FPG (P_interaction_ = 0.011) and HbA1c (P_interaction_ = 0.007). Among individuals with lower intake of plant protein (<39 g/day), those with >1 risk allele had higher FPG (*p* = 0.001) and HbA1c (*p* = 0.00006) than individuals with ≤1 risk allele (Figure 1). Furthermore, among individuals with higher intake of animal protein (>19 g/day), those with >1 risk allele had higher FPG (*p* = 0.008) and HbA1c (*p* = 0.001) than individuals with ≤1 risk allele (Figure S2). None of the interactions were significant between the 7-SNP GRS and dietary intakes on metabolic traits except for the interactions between 7-SNP GRS and protein intake on HbA1c (P_interaction_ = 0.032), and 7-SNP GRS and carbohydrate intake (P_interaction_ = 0.04) on fasting insulin. However, these interactions were not significant after stratifying based on animal and plant protein.

### 3.4. Sensitivity Analyses

We subjected our regression results to a wide range of robustness checks. First, we adjusted for duration of diabetes, and the association of 3-SNP GRS with HbA1c and FPG (*p* = 0.010 and 0.040, respectively) and the interaction of 3-SNP GRS with protein intake (%) (P_interaction_ = 0.025 and 0.019 for HbA1c and FPG, respectively) were still significant. Second, we excluded individuals with diabetes, and this resulted in a small sample size of 496 NGTs. However, a significant association of 3-SNP GRS with HbA1c (*p* = 0.012) was still observed, but none of the interactions were statistically significant (*p* = 0.126 and 0.405 for HbA1c and FPG, respectively). Third, given the association between dietary fat intake and T2D traits, we adjusted for total dietary fat intake and found that the interaction of 3-SNP GRS with protein intake (%) (P_interaction_ = 0.007 and 0.009 for HbA1c and FPG, respectively) was still significant. Fourth, we tested for the interaction between 3-SNP GRS and dairy protein intake to see if the interactions with the animal protein intake were driven by the intake of dairy protein. We found that the interactions between 3-SNP GRS and dairy protein intake were not statistically significant (P_interaction_ = 0.439 and 0.597 for HbA1c and FPG, respectively), suggesting that dairy protein intake is unlikely to confound the GRS–animal protein intake interaction on diabetes traits. Fifth, in addition to the aforementioned factors, we adjusted for other possible confounders such as physical activity level, smoking, alcohol consumption and fibre intake, and found that the interactions between the 3-SNP GRS and protein intake on HbA1c and FPG were still significant (P_interaction_ = 0.009 and 0.008, on HbA1c and FPG, respectively).

## 4. Discussion

The current research provides evidence for the GRS–protein intake interaction on T2D-related traits in Asian Indians. We found that individuals with >1 risk allele had higher FPG and HbA1c levels than those with ≤1 risk allele among individuals with lower intake of plant protein (<39 g/day). Given that the prevalence of obesity, high FPG and T2D has increased in India from 1990 to 2016 [55], our findings are of importance in terms of public health. Our study suggests that increasing the intake of plant protein might be an effective strategy towards better management of blood glucose levels, especially in Asian Indians with a higher genetic susceptibility for T2D.

In the present study, the 3-SNP GRS was associated with higher WC, which is in accordance with the findings in 7067 individuals from the Indian Migrant Study where a combined risk score of eight variants was observed to be nominally associated with higher WC (*p* = 0.02) [56]. The 3-SNP GRS was also associated with FPG and HbA1c, where individuals with higher GRS had higher FPG and HbA1c. Similarly, a large GWAS in 159,940 individuals of African, South Asian, East Asian and European ancestries identified 60 genetic variants influencing HbA1c [57], including SNPs located in the *FTO* and *TCF7L2* genes. An association of 8-SNP GRS with T2D was found in a case-control study of 5,148 Indians (including 1808 individuals with T2D and 1549 controls) from in and around Pune in western India [25]. A case-control study of 3357 Indian adults (including 2486 individuals with T2D and 2678 controls) also found that individuals with a higher GRS, derived from 32 SNPs, were at a higher T2D risk compared to those with lower GRS [58]. The EpiDREAM prospective cohort study (*n* = 15,466 individuals) has shown that South Asians might have a greater genetic load for T2D than Latinos and Europeans [59]. If our study findings are confirmed in larger cohorts, our 3-SNP GRS might serve as a diagnostic marker for investigating the cumulative effect of SNPs on diabetes-related traits and identifying Asian Indians with a high genetic risk of T2D.

Increasing evidence has shown that certain dietary factors might interact with genetic susceptibility in relation to the risk of diabetes and related traits [40,41,42,44,60]. In our study, individuals with higher 3-SNP GRS had higher fasting glucose and HbA1c concentrations than individuals with lower GRS among individuals with lower intake of plant protein. The results of the current analysis are in agreement with a recent study among Southeast Asian women (*n* = 110) showing significant interactions between a 15-SNP GRS and total protein intake. The study found that consuming a low protein diet (13.51 ± 1.18% of TEI) was associated with lower WC and triacylglycerol concentrations, particularly in individuals with high genetic risk [60]. Moreover, significant interactions of the *FTO* SNPs (rs8044769 (C>T), rs3751812 (G>T) and rs8050136 (A>C)) with protein intake on blood glucose were observed in 819 Polish adults, where higher protein intake (>18% of TEI) was associated with higher blood glucose in individuals with the TT genotype of rs8044769, CC genotype of rs8050136 and GG genotype of rs3751812 [61]. However, the effect of protein sources was not analysed in the abovementioned studies, thus a direct comparison between these studies and our findings cannot be performed. In contrast to our study, a large prospective case-cohort study from eight European countries (*n* = 21,900) found no significant interactions between intake of protein and metabolic GRSs on T2D [62]. Similarly, no interaction was found between protein intake and a 10-SNP GRS on T2D risk among 8842 Korean adults [42]. These discrepancies in the findings might be due to differences in ethnicity, dietary assessments, dietary patterns, relative proportions of different macronutrients, protein sources, sample sizes and GRS construction methods; hence, larger studies in multiple ethnic groups are needed to confirm the GRS–protein intake interactions.

Previous studies have examined the relationship between protein intake and T2D in South Indians. A cross-sectional study of 900 urban South Indians from Chennai demonstrated that individuals with known T2D had significantly higher protein intake (15.9%) than controls (14%) [63]. Another study in Asian Indians from different parts of India reported similar findings, where diabetic individuals (*n* = 385) had higher protein intake (14%) than controls (12%) (*n* = 409) [64]. A cohort including 146 Asian Indians living in San Francisco found that individuals were at increased T2D risk when the protein intake was high. The same study also reported that the intake of animal protein (32 ± 15 g/day) was more likely to be associated with diabetes risk (*p =* 0.07) in comparison with the intake of vegetable protein (38 ± 8 g/day; *p =* 0.26) [65]. Even though consuming diets high in protein has been one of the most popular strategies for losing weight and the management of overweight and obesity [66,67,68], the health impacts of diets high in protein on T2D are inconsistent. Higher animal protein intake, but not plant protein, showed significant association with a higher risk of T2D in 38,094 individuals (median intake of animal protein = 62 g/day; 10-year follow-up period) from the European Prospective Investigation into Cancer and Nutrition-Netherlands (EPIC-NL) study [13], and in 37,309 women from the US (median intake of total meat in the highest quintiles = 53.5 serving/day; 8.8 year follow-up period) from the Women’s Health Study [14]. Moreover, a large case-cohort study including 28,557 European individuals reported that higher animal protein intake was associated with higher incidence of T2D (per 10 g: 1.05 (1.02–1.08), P_trend_ = 0.001) over an average follow-up period of 12 years [16]. Furthermore, the higher intake of animal protein (5% increase in consumption of protein derived from meat and meat products) was shown to be associated with a 34% increased risk of T2D, whereas the intake of plant protein was shown to have a considerable protective effect in 1190 elderly participants from the Mediterranean islands [17]. A large study of 92,088 women and 40,722 men from the United States found that substituting 5% of energy intake from animal protein with plant protein was associated with a decrease in T2D risk by 23% [69]. Moreover, a systematic review and meta-analysis of thirteen randomised controlled trials (*n* = 280 middle-aged adults from Iran, Denmark, United States, Germany, Canada and Greece) found significant decreases in HbA1c, fasting insulin and fasting glucose in diets that substituted animal protein with plant protein at a median level of ~35% of total protein intake/day [70]. Another systematic review and meta-analysis of eleven cohort studies, including individuals from the United States, Europe, Asia, Melbourne and Finland (52,637 cases among 483,174 individuals)**,** showed that the intakes of total protein and animal protein increased T2D risk in both men and women, whereas plant protein intake decreased T2D risk in women [71]. Previous cohort studies in the United States (90,239 women and 40,539 men) and in the Netherlands (6798 individuals) found that an association between the higher adherence to a plant-based diet and a lower risk of T2D [72,73]. In contrast, other prospective cohort studies (*n* = 8370–38,094 individuals) observed no significant associations [13,74,75]. It is possible that the interactions between genetic factors and protein intake might be one of the reasons for the discrepancies in the effect of dietary protein intake on the risk of T2D and its related traits.

The dietary patterns across different parts of India have been significantly affected by urbanisation. Given that food availability and purchasing power are higher in urban than rural areas, diets of both residents tend to differ significantly [12,76]. Protein intake has been shown to be positively related to an individual’s income, where the demand for animal protein increased with the disposable income [12]. Higher protein intake has also been reported in urban areas in India, with the overall mean intake of protein being the highest in the high-income group (73.1 g/day) followed by the middle-income group (63.2 g/day), industrial labourer (59.4 g/day) and low-income group (57.8 g/day) [77,78]. The present study included urban residents and the mean protein intake is 71.6 ± 22.7 g/day, which is higher than dietary protein recommendations for Asian Indians (55–60 g/day) [79]. However, the mean protein intake is only 11% (percentage calories coming from the protein), which is similar to the previous large studies, such as the National Family Health and National Nutrition Monitoring Bureau surveys that were conducted in the Indian population [80,81]. A study in 6907 adults from South India aged >20 years showed that the consumption of pulses was lower in rural compared to urban Indian adults [82] and a cross-sectional study including 56,742 men and 99,574 women aged 20–49 years also demonstrated that inverse association between daily or weekly legumes and the presence of diabetes [83]. A recent study in 1033 Indian adults also showed that a significant decrease in the risk of T2D was observed among those having higher intakes of legumes and pulses [84]. In the same population, a study in 2042 individuals reported that pulses and legumes contributed to only 17.2% of the daily protein suggesting a reduced intake of plant protein [85]. Hence, according to the findings from the previous studies and the GRS–plant protein intake interaction from the present study, increasing the intake of plant protein might be an effective strategy to arrest the rising epidemic of T2D among Indian adults.

The strength of this study is the use of a representative sample of the urban Chennai population. Given that diabetes prevalence continues to be higher in urban residents compared to rural residents in India [2,86,87], understanding gene–diet interactions on T2D in urban areas would improve diabetes prevention strategies among urban Indians. Our study used unweighted GRSs to analyse the combined effect of several SNPs, which is an effective approach to study polygenic diseases such as T2D and obesity, providing a better knowledge of disease risk compared to a single-SNP analysis [36]. A comprehensive and validated semi-quantitative FFQ was used for analysing dietary intakes [48]. Furthermore, anthropometric outcomes were assessed by qualified staff rather than self-reported to improve the accuracy of anthropometric measurements. However, the study has several limitations. First, the study has a small sample size suggesting that we might have had insufficient power for our analysis. To maximise power, we used a GRS approach, which has an advantage over single-SNP analysis, and significant associations and interactions were found. Second, the observational nature of the study design cannot explain causal relationships or exclude residual confounding; however, sensitivity analyses were carried out where adjustment was performed for additional confounding factors such as diabetes duration, total fat intake, physical activity level, anti-diabetic medication, alcohol consumption, smoking and fibre intake. Third, dietary intake was assessed using self-reported FFQ, which might have introduced recall and measurement bias. Finally, SNPs contributing to our GRSs represent only a small proportion of the increasing number of identified metabolic disease-associated variants in Asian Indians; however, we have chosen SNPs in *TCF7L2* and *FTO* genes that have presented the most consistent and strongest associations with T2D and obesity, respectively, in several populations [32,88].

## 5. Conclusions

In summary, the current study has found a novel GRS–protein intake interaction where individuals with >1 risk allele and lower intake of plant protein (<39 g/day) had higher FPG and HbA1c levels. This suggests that increasing the intake of plant protein may be an effective approach to overcome the genetic risk of diabetes in urban Asian Indians. To prove this hypothesis, appropriate randomised clinical trials with diets of higher and lower plant protein intake need to be performed. Moreover, there is a need for studies with larger sample sizes to confirm gene–diet interactions. Ultimately, there is a need for the assessment of the clinical benefit of targeted interventions based on an individual’s underlying genetic risk.

## Figures and Tables

**Table 1:** Characteristics of study participants.

	Total		NGT Controls		T2D Cases		*p* Value
	*n*		*n*		*n*		
Sex							0.807 **
Men (%)	591	56	278	56	313	55	
Women (%)	471	44	218	44	253	45	
Age (years)	1062	45 ± 12	496	38 ± 10	566	51 ± 11	1.160 × 10^−71^ *
Diabetes duration	-	-	-	-	566	5.20 ± 5.29	-
Anti-diabetic medication	-	-	-	-	164	15.4%	-
BMI (kg/m^2^)	1061	24.6 ± 4.56	496	23.5 ± 4.64	565	25.5 ± 4.30	1.480 × 10^−12^ *
WC (cm)	1022	87 ± 12	479	83 ± 12	543	91 ± 10	5.692 × 10^−33^ *
HBA1C (%)	1056	7.3 ± 2.4	492	5.6 ± 0.47	564	8.8 ± 2.4	1.480 × 10^−14^ *
FPG (mg/dL)	1060	126 ± 64	495	85 ± 8	565	162 ± 69	1.392 × 10^−127^ *
Fasting Insulin (μIU/mL)	699	9 ± 7	448	8 ± 6	251	12 ± 7	6.386 × 10^−101^ *
Energy (kcal/day)	1062	2536 ± 805	496	2685 ± 708	566	2406 ± 861	8.773 × 10^−9^ *
Protein (%)	1062	11 ± 1	496	11.27 ± 1.17	566	11.45 ± 1.23	0.014 *
Animal protein (g/day)	1062	22 ± 12	496	25 ± 13	566	19 ± 11	3.787 × 10^−14^ *
Plant protein (g/day)	1062	40 ± 14	496	42 ± 15	566	39 ± 13	0.006 *
Fat (%)	1062	23 ± 5	496	24 ± 5	566	23 ± 5	0.113 *
Carbohydrate (%)	1062	65 ± 6	496	64 ± 6	566	65 ± 6	0.003 *
Dietary fibre (g)	1062	32 ± 11	496	32 ± 10	566	31 ± 12	0.150 *
Total SFA (g)	1062	24 ± 10	496	27 ± 10	566	22 ± 10	2.295 × 10^−12^ *
Total MUFA (g)	1062	20 ± 8	496	21 ± 8	566	18 ± 8	3.943 × 10^−9^ *
Total PUFA (g)	1062	18 ± 10	496	19 ± 9	566	18 ± 10	0.184 *
Physical activity level							
Sedentary	695	71%	335	73%	360	70%	0.001 **
Moderate	223	23%	110	24%	113	22%	
Vigorously active	58	6%	13	3%	45	8%	
Smoking							0.206 **
Non-smokers	865	81.5%	396	79.8%	469	82.9%	
Smokers	197	18.5%	100	20.2%	97	17.1%	
Alcohol consumption							
Non-alcoholics	793	74.7%	358	72.2%	435	76.9%	0.080 **
Alcoholics	269	25.3%	138	27.8%	131	23.1%	

Data presented as Mean ± SD. *p* values are for the mean differences between controls and T2D cases using an independent sample *t*-test. ** *p* values are from the Chi-squared test. Frequency of men and women between controls and cases was compared using a chi-square test. Abbreviations: NGT, normal glucose tolerance; T2D, type 2 diabetes; BMI, body mass index; WC, waist circumference; HbA1c, glycated haemoglobin; FPG, fasting plasma glucose; SFA, saturated fatty acids; MUFA, monounsaturated fatty acids; PUFA, polyunsaturated fatty acids.

**Table 2:** Associations of 7-SNP and 3-SNP GRS and with metabolic traits.

	7-SNP GRS					3-SNP GRS				
	*n*	GRS < 6	*n*	GRS ≥ 6	*p* Value	*n*	GRS ≤ 1	*n*	GRS > 1	*p* Value *
BMI (kg/m^2^)	526	24.5 ± 0.2	535	24.7 ± 0.2	0.572	645	24.7 ± 0.2	416	24.5 ± 0.2	0.572
WC (cm)	508	86.7 ± 0.5	514	87.4 ± 0.5	0.668	620	87.0 ± 0.47	402	88.0 ± 0.57	0.010
HBA1C (%)	524	7.1 ± 0.1	532	7.4 ± 0.1	0.935	640	7.0 ± 0.1	416	7.7 ± 0.1	0.000066
FPG (mg/dL)	526	119.9 ± 2.6	534	131.6 ± 2.9	0.181	644	120.0 ± 2.35	416	135.0 ± 3.39	0.002
Fasting insulin (μIU/mL)	373	9.5 ± 0.4	326	9.4 ± 0.3	0.767	419	10.0 ± 0.36	280	9.0 ± 0.33	0.171

Data are Mean ± standard error of the mean. * *p* values adjusted for sex, age, T2D, anti-diabetic medication and additionally for BMI, when BMI is not an outcome. The analysis was carried out using log-transformed variables. Abbreviations: GRS, genetic risk score; BMI, body mass index; WC, waist circumference; HbA1c, glycated haemoglobin; FPG, fasting plasma glucose.

**Table 3:** Association of 7-SNP and 3-SNP GRSs with T2D.

GRS	OR	95% CI for OR		*p* Value *
		Lower	Upper	
7-SNP GRS	2.083	1.496	2.898	0.0000134
3-SNP GRS	1.559	1.121	2.170	0.008

* *p* values were obtained from the logistic regression models adjusted for sex, age, anti-diabetic medication and BMI. Abbreviations: GRS, genetic risk score; SNP, single nucleotide polymorphism; T2D, type 2 diabetes; OR, odds ratio; CI, confidence interval; BMI, body mass index.

**Table 4:** Interactions of 7-SNP and 3-SNP GRSs with dietary factors on metabolic traits.

	7-SNP GRS			3-SNP GRS		
	Protein	Fat	Carbohydrate	Protein	Fat	Carbohydrate
	(% of TEI)	(% of TEI)	(% of TEI)	(% of TEI)	(% of TEI)	(% of TEI)
**BMI (kg/m^2^)**	0.176	0.388	0.195	0.36	0.653	0.805
**WC (cm)**	0.852	0.786	0.892	0.638	0.958	0.914
**HBA1C (%)**	0.032	0.629	0.618	0.007	0.677	0.756
**FPG (mg/dL)**	0.249	0.489	0.507	0.011	0.367	0.231
**Fasting insulin (μIU/mL)**	0.952	0.085	0.04	0.299	0.567	0.999
**T2D**	0.956	0.214	0.152	0.764	0.508	0.365

Data are P_interaction_ values adjusted for sex, age, T2D, antidiabetic medications and additionally for BMI, when BMI is not an outcome. The analysis was carried out using log-transformed variables. Abbreviations: GRS, genetic risk score; TEI, total energy intake; BMI, body mass index; WC, waist circumference; HbA1c, glycated haemoglobin; FPG, fasting plasma glucose; T2D, type 2 diabetes.

## Supplementary Materials

The following are available online at https://www.mdpi.com/article/10.3390/nu13093064/s1, Table S1: Genotype distribution of the seven SNPs that were chosen for our study (*n* = 1062), Figure S1: Methodology of the Chennai Urban Rural Epidemiology Study (CURES), Figure S2: Interaction between 3-SNP GRS and animal protein intake (%) on fasting plasma glucose and glycated haemoglobin after adjusting for anti-diabetic medication.
