# Supplementary material for: Lower Dietary Intake of Plant Protein Is Associated with Genetic Risk of Diabetes-Related Traits in Urban Asian Indian Adults
Source: Nutrients. 2021 Aug 31;13(9):3064. doi: 10.3390/nu13093064 (PMC8466015; doi:10.3390/nu13093064)
Supplement: Supplementary file 1 [file nutrients-13-03064-s001.zip › nutrients-1281838-supplementary.pdf]

Supplementary information

Table S1. Genotype distribution of the seven SNPs that were chosen for our study (n= 1,062)

Gene	SNP	MAF among NGTs	MAF among T2D cases	Overall MAF	HWE
TCF7L2	rs12255372	0.20	0.21	T= 0.21	0.36
TCF7L2	rs7903146	0.30	0.33	T= 0.31	0.12
FTO	rs8050136	0.06	0.13	A= 0.10	0.35
FTO	rs918031	0.49	0.48	C= 0.49	0.09
FTO	rs1588413	0.25	0.32	T= 0.29	0.15
FTO	rs11076023	0.49	0.45	T= 0.47	0.48
FTO	rs7193144	0.13	0.12	T= 0.12	0.16

Abbreviations: SNP single nucleotide polymorphisms; MAF minor allele frequency; T2D type 2 diabetes; NGT, Normal Glucose Tolerance; HWE Hardy-Weinberg equilibrium; *TCF7L2* Transcription factor 7-like 2; *FTO* fat mass and obesity-associated.

Figure S1: Methodology of the Chennai Urban Rural Epidemiology Study (CURES)

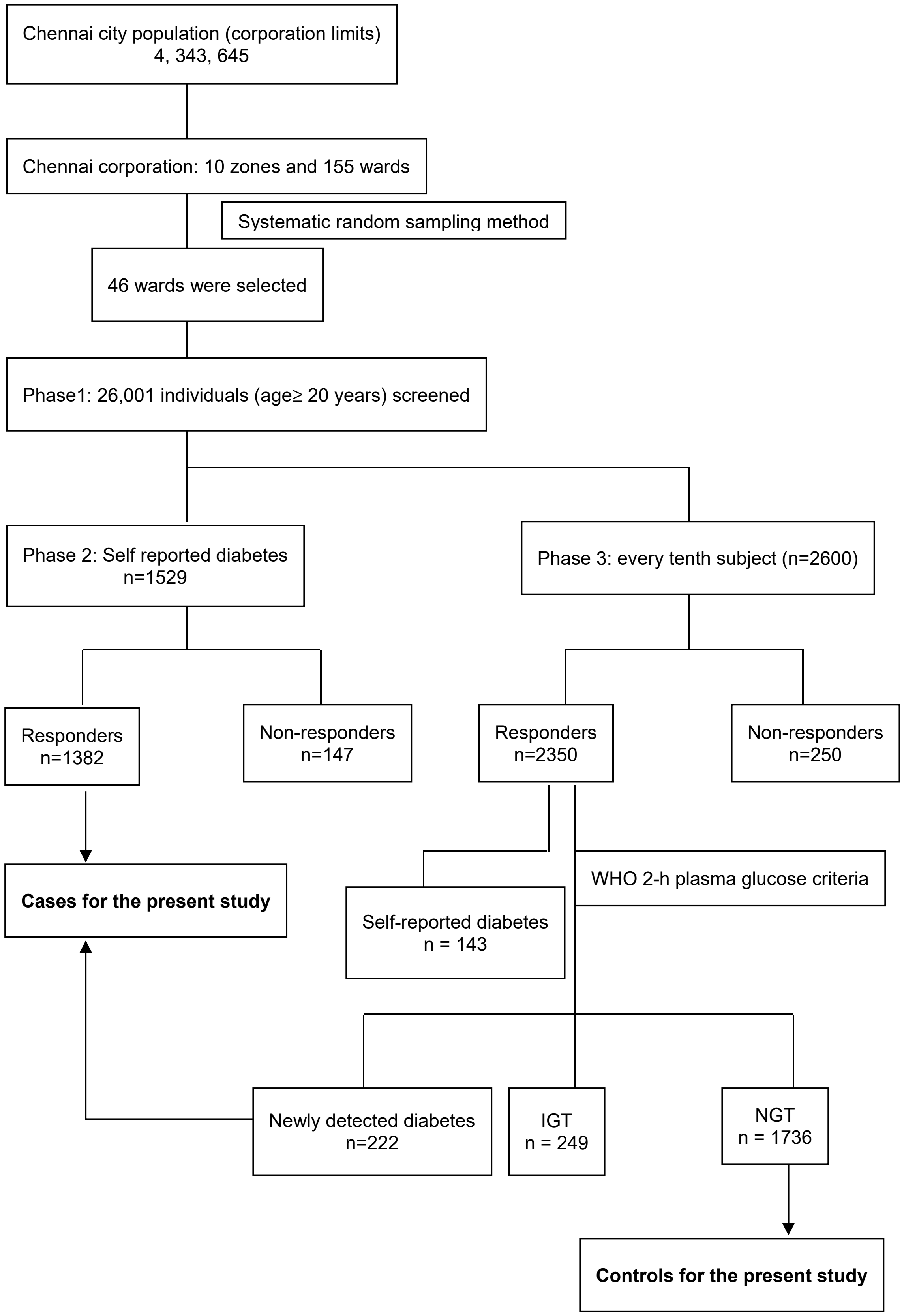

Figure S2: **Interaction between 3-SNP GRS and animal protein intake (%) on fasting plasma glucose and glycated haemoglobin after adjusting for antidiabetic medications**. White bars indicate individuals with GRS ≤1 risk allele; the black bars indicate individuals with GRS >1 risk allele. (a) Individuals with>1 risk allele had a significantly higher FPG compared to those with ≤1 risk allele, among those with higher intake of animal protein (>19 g/day) (P=0.008). (b) Individuals with>1 risk allele had a significantly higher HbA1c compared to those with ≤1 risk allele, among those with higher intake of animal protein (>19 g/day) (P=0.001). P values were adjusted for age, sex, T2D, BMI, antidiabetic medications, total fat intake (%) and TEI. Abbreviations: GRS, genetic risk score; FPG, fasting plasma glucose; HbA1c, glycated haemoglobin and TEI, total energy intake.

(a)

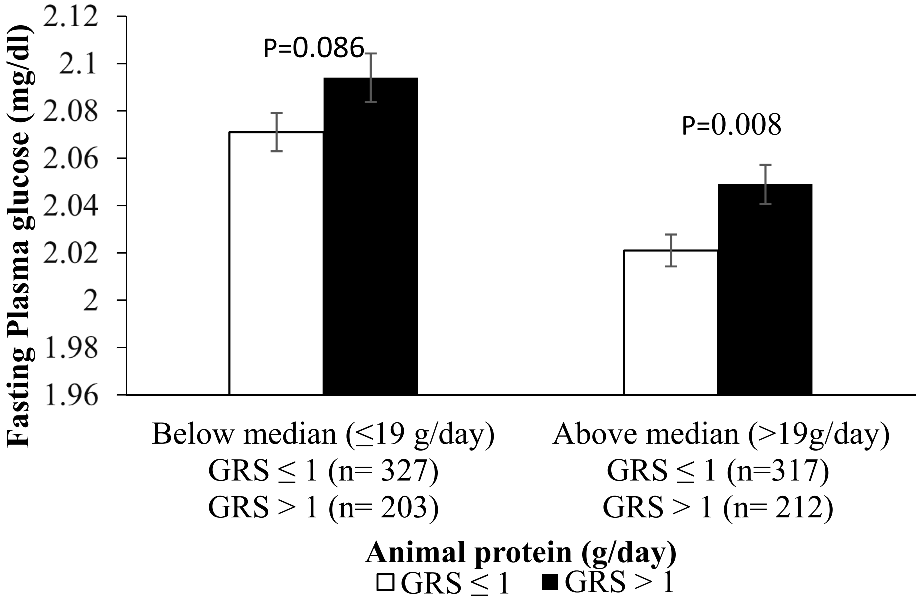

(b)

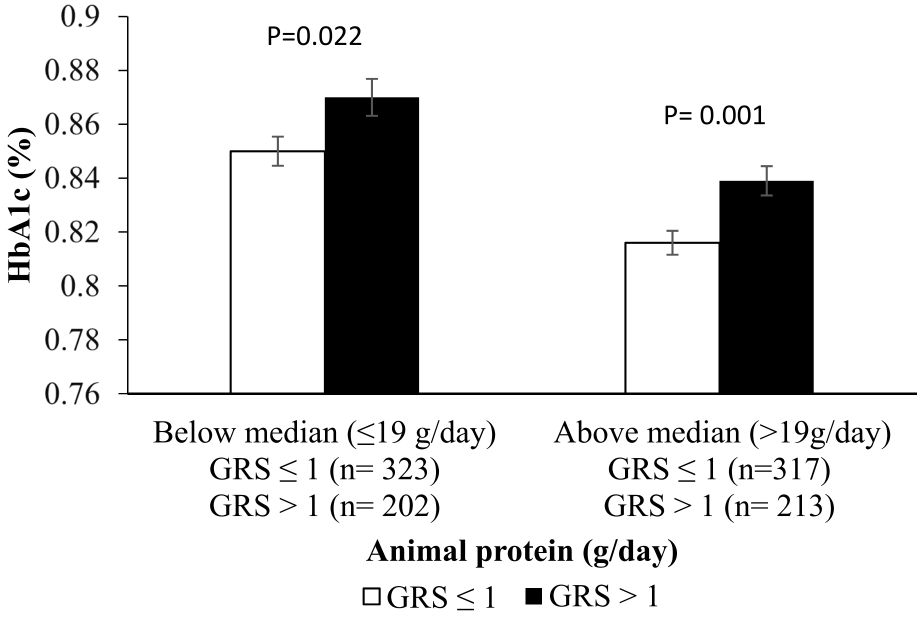

P=0.022
P= 0.001
HbA1c (%)
0.9
0.88
0.86
0.84
0.82
0.8
0.78
0.76
Below median (≤19 g/day)
GRS ≤ 1 (n= 323)
GRS > 1 (n= 202)
Above median (>19g/day)
GRS ≤ 1 (n=317)
GRS > 1 (n= 213)
Animal protein (g/day)
GRS ≤ 1
GRS > 1
